# Identification and differential regulation of microRNAs during thyroid hormone-dependent metamorphosis in *Microhyla fissipes*

**DOI:** 10.1186/s12864-018-4848-x

**Published:** 2018-06-28

**Authors:** Lusha Liu, Wei Zhu, Jiongyu Liu, Shouhong Wang, Jianping Jiang

**Affiliations:** 1 Chengdu Institute of Biology, Chinese Academy of Sciences, Chengdu, 610041 China; 2 University of Chinese Academy of Sciences, Beijing, 100049 China

**Keywords:** TH, miRNA, Metamorphosis, Morphology, Histology, *Microhyla fissipes*, Target gene

## Abstract

**Background:**

Anuran metamorphosis, which is obligatorily initiated and sustained by thyroid hormone (TH), is a dramatic example of extensive morphological, biochemical and cellular changes occurring during post-embryonic development. Thus, it provides an ideal model to understand the actions of the hormone and molecular mechanisms underlying these developmental and apoptotic processes. In addition to transcriptional factors, microRNAs (miRNAs) play key roles in diverse biological processes via post-transcriptional repression of mRNAs. However, the possible role of miRNAs in anuran metamorphosis is not well understood. Screening and identification of TH-responding miRNAs are required to reveal the integrated regulatory mechanisms of TH during metamorphosis. Given the specific role of TRs during *M. fissipes* metamorphosis and the characteristics of *M. fissipes* as an ideal model, Illumina sequencing technology was employed to get a full scope of miRNA in *M. fissipes* metamorphosis treated by T3.

**Results:**

Morphological and histological analysis revealed that 24 h T3 treatment *M. fissipes* tadpoles resembled that at the climax of natural metamorphosis. Thus, small RNA libraries were constructed from control and 24 h T3 treatment groups. A total of 164 conserved miRNAs and 36 predicted novel miRNAs were characterized. Furthermore, 5′ first and ninth nucleotides of miRNAs were significantly enriched in U in our study. In all, 21 miRNAs were differentially expressed between the T3 and control groups (*p* < 0.01). A total of 10,206 unigenes were identified as target genes of these differentially expressed miRNAs. KEGG pathway analysis indicated that the most overrepresented miRNA target genes were enriched in the “PI3k-Akt signaling pathway”. In addition, a network associated with the TH signaling pathway provides an opportunity to further understand the complex biological processes that occur in metamorphosis.

**Conclusions:**

We identified a large number of miRNAs during *M. fissipes* metamorphosis, and 21 of them were differentially expressed in the two groups that represented two different metamorphic stages. These miRNAs may play important roles during metamorphosis. The study gives us clues for further studies of the mechanisms of anuran metamorphosis and provides a model to study the mechanism of TH-affected biological processes in humans.

**Electronic supplementary material:**

The online version of this article (10.1186/s12864-018-4848-x) contains supplementary material, which is available to authorized users.

## Background

Amphibian metamorphosis is a dynamic post-embryonic development phase that adapts aquatic to terrestrial life. Thyroid hormone (TH: T3 and T4) induces the complete metamorphosis of anuran tadpoles into juvenile frogs, which resembles mammalian postembryonic development around birth in the presence of high levels of TH [1]. Morphological changes induced by TH cover organogenesis such as limb formation, complete organ degeneration such as gill and tail degeneration [2], and comprehensive organ remodeling such as intestine, pancreas, liver, dorsal muscles and brain remodeling [3, 4]. Whereas it is difficult to manipulate uterus-enclosed mammalian embryos, tadpoles are easily manipulated and independent of any maternal influence [5]. Therefore, anuran metamorphosis regulated by TH provides an ideal model for studying the mechanisms underlying hormone-responsive morphological and functional remodeling, tissue-specific responses to hormone, the precise timing of morphological changes, and the development of adult organ-specific stem cells in vertebrates. Furthermore, anuran postembryonic development, which is triggered and orchestrated by TH, serves as a powerful model to identify the effects of environmental chemical contaminants on TH signaling in vertebrates.

Metamorphosis can be divided into three periods based on the sequence of developmental processes: premetamorphosis, where tadpoles grow and develop hindlimb buds; prometamorphosis, where hindlimbs develop and digits differentiate; and metamorphic climax, where forelimbs emerge and the tail is resorbed completely [6]. A significant number of researches have been devoted to study the mechanisms of anuran metamorphosis induced by THs. THs participate in cellular signal transduction by acting as the ligands of thyroid hormone receptors (TRs) [3]. When TH is absent, the unliganded TR/9-cis-retinoic acid receptor (TR/RXR) heterodimers recruit the co-repressors complex that has the histone deacetylase (HDAC) 3 activity [7]. Histone deacetylation leads to a closed chromatin conformation that is inaccessible for transcriptional machinery, thus repressing the expression of downstream genes. When liganded with TH, a conformational change in the TR induces it to release the co-repressor complex and recruit an SRC/p300 co-activator complex that contains histone acetyl transferase activity [6, 8]. These histone modifications result in chromatin opening and the activation of direct TH response genes expression. TRs can also recruit co-activator complexes such as the SWI/SNF complex, which is involved in chromatin remodeling and the Mediator complex directly involved in transcription activation [9]. In turn, the products of these direct target genes affect the expression of downstream genes. A large number of genes and pathways activated by TH in the larval amphibian during metamorphosis have been identified [10–15]. Particularly, TH directly upregulates the expression of Shh and induces bone morphogenetic protein-4 (BMP-4) expression in the *Xenopus laevis* intestine during metamorphosis, which indicates that the Shh/BMP-4 signaling pathway plays key roles in amphibian intestinal remodeling [16]. Notch signaling plays a role in stem cell development by regulating the expression of Hairy genes during intestinal remodeling in metamorphosis [5]. Two key members of the mitochondrial death pathway, *XR11* and *caspase-9*, are found to regulate the proliferative status of neuroblasts in the *Xenopus* brain during metamorphosis [17]. Therefore, TH alters a number of genes and regulatory pathways leading to morphological changes during metamorphosis.

In addition to transcriptional factors, microRNAs (miRNAs) play key roles in the regulation of post-transcriptional gene expression during diverse biological processes such as cell proliferation, programmed cell death, fat metabolism, organ development, tumorigenesis and behavior [18]. MiRNAs are endogenous, approximately 22 nucleotides (nt), small non-coding RNA molecules that regulate gene expression via complementary binding to the 3′ untranslated regions (UTRs) of target messenger RNAs (mRNAs) and causing mRNA cleavage or translation blockage [19, 20]. Previous studies have shown that miRNAs are involved in *Xenopus* oocyte, egg, early embryo, liver, and skin development [20–23]. Given that miRNAs play key roles in regulating gene expression during anuran development, identifying miRNAs and their target genes that are responsible for amphibian metamorphosis is particularly meaningful. Although a series of genes, signaling pathways and even hormones related to metamorphosis have been identified previously, miRNAs involved in anuran metamorphosis, as well as their potential functional networks, are still less reported. Only 75 unique miRNAs belonging to 30 conserved families in *Xenopus tropicalis* have been identified during anuran metamorphosis so far [24]. Although some effects of miRNAs on metamorphosis have been clarified in these studies, the specific functions of miRNAs and the complicated molecular regulatory networks they may participate in remain unclear. Therefore, our limited knowledge of miRNAs related to anuran metamorphosis prevents us from having a systematic and common understanding of the miRNA:mRNA regulation of anuran metamorphosis.

*Microhyla fissipes* is a small-sized and widely distributed anuran in southeastern Asia and eastern Asia. Due to its fast development, transparent tadpole phase, biphasic life cycle, diploid, and embryo development in vivo, *M. fissipes* is a good model to study tissue development and apoptosis, environmental toxicology, and adaptive mechanisms from aquatic to terrestrial life [25].

Given the specific role of TRs during *M. fissipes* metamorphosis and the characteristics of *M. fissipes* as an ideal model [6, 25], Illumina sequencing technology was employed to obtain a full scope of miRNAs in *M. fissipes* metamorphosis induced by T3. From these sequencing data and the subsequent miRNA annotation, we identified differentially expressed miRNAs that may play important roles in regulating metamorphosis initiation and the cellular process during metamorphosis. The identification and characterization of known and novel miRNAs will enable us to better understand the roles of these miRNAs in anuran metamorphosis. Subsequently, we predicted the mRNA targets of *M. fissipes* miRNAs and correlated their expression with the mRNA expression identified in a previous study [6]. The integrated analysis of miRNA and mRNA expression profiles will explore their possible regulatory patterns at the critical stage of anuran development. This study will advance our understanding of how post-transcriptional regulators may regulate TH dependent metamorphosis and will provide a model to study the mechanisms how TH affects such biological processes in humans.

## Methods

### Tadpoles

Mature female and male *M. fissipes* were collected from Shuangliu, Chengdu, China (30.5825^o^ N, 103.8438^o^ E) in June, 2016. The care and treatment of animals in this study were performed according to the Guideline for the Care and Use of Laboratory Animals in China, and the animal experiments were approved by the Experimental Animal Use Ethics Committee of the Chengdu Institute of Biology (Permit Number: 2016036). After acclimatized in our laboratory for 1 week, the male and female were injected LHRHa with 0.3 μg/g body weight resolving dosage. Embryos were obtained from one pair of frogs and were subjected to a 12:12 h light:dark cycle at 25 ± 0.6 °C. Tadpole developmental stage was recorded using the *M. fissipes* developmental table [26].

### Sample collection

Tadpoles at stage 33 (premetamorphosis, oar-shaped limb bud) were exposed to 10 nM 3,3′,5-triiodo-l-thyronine sodium (T3, Sigma). The chemicals were renewed every 12 h of exposure when the medium was refreshed. At each exposure time point (0 h, 12 h, 24 h, 36 h, and 48 h), six tadpoles were randomly collected for morphological and histological observations. Tadpoles treated for 0 h were set as the control group. For the small RNA library construction, samplings of the control group (Control-1, Control-2, and Control-3) and the T3 exposure group (24 h) (T3–1, T3–2, and T3–3) had three biological replicates, respectively, and each replicate was made up of one individual. Tadpoles in this study were anesthetized in 0.01% MS222 (ethyl 3-aminobenzoate methanesulfonate) and were then processed as described below. Each individual from the control and T3 groups for small RNA sequencing was immediately immersed in liquid nitrogen and stored at − 80 °C until the RNA was extracted (within 3 days).

### Morphological and histological observations of tadpoles

**Fig. 1:**
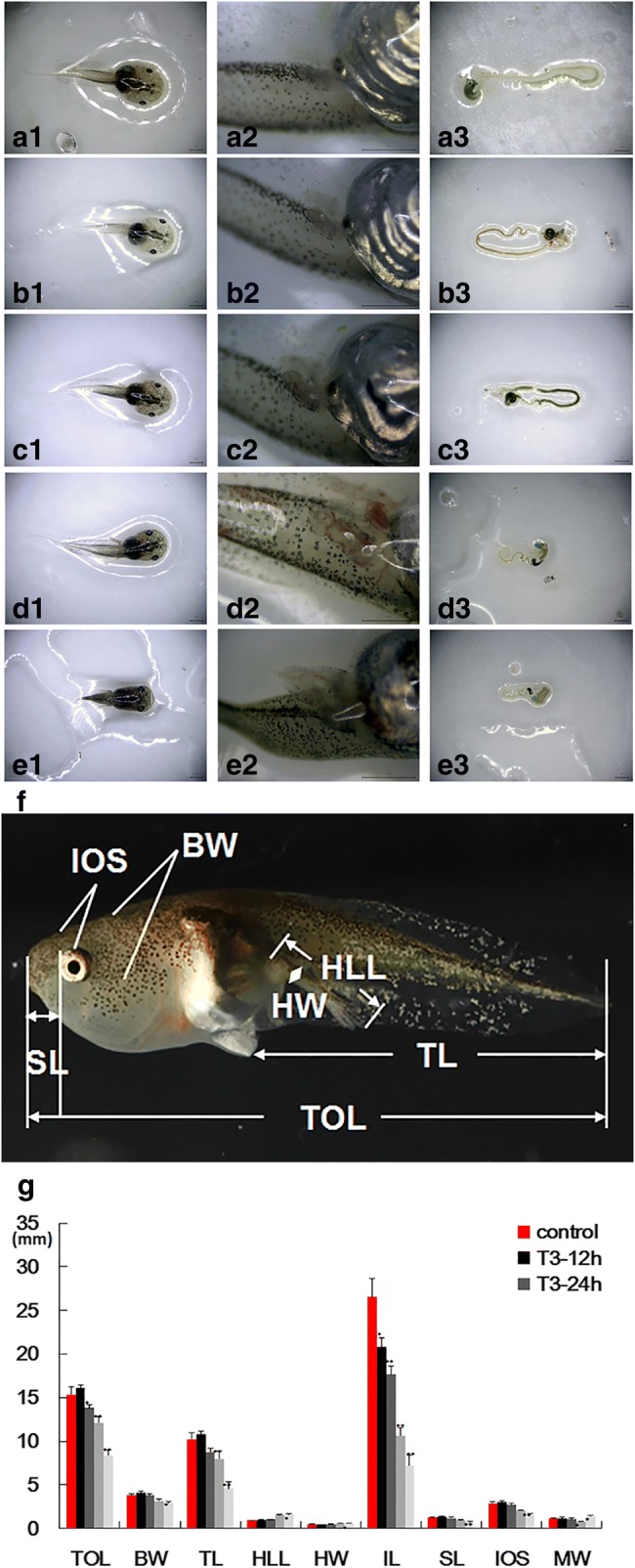
**a**-**e** Morphological changes of *M. fissipes* tadpoles induced by T3. **a1**-**a3**: control; **b1**-**b3**: T3–12 h; **c1**-**c3**: T3–24 h; **d1**-**d3**: T3–36 h; **e1**-**e3**: T3–48 h. Scale bar = 1 mm. **f** Morphological parameter of *M. fissipes* tadpoles. TOL: total length; TL: tail length; BW: body width; HLL: hind limb length; HW: hindlimb width; SL: snout length; IOS: interocular space. **g** Morphological parameter of *M. fissipes* tadpoles treated with T3 at different time points. Bars represent the mean ± SD (*n* = 6). The data were analyzed by One-Way ANOVA and significance is set at *p* < 0.05. The asterisks indicate a significant difference compared to control, *p < 0.05, **p < 0.01. IL: intestine length

A stereo microscope (JSZ8T, Jiang Nan Yong Xin, Nanjing, China) with the Mshot Image Analysis system (Mc50-N) was used to observe and take photos of tadpoles and to measure their total length (TOL), tail length (TL), body width (BW), hindlimb length (HLL), hindlimb width (HW), snout length (SL), interocular space (IOS) and mouth width (MW) (Fig. 1). After external morphological observations, intestines were obtained for measuring intestine length (IL). Then, the small intestines were dissected and were fixed in Bouin’s Fluid. After dehydration in a graded series of ethanol and transparency by xylene, intestines were embedded in paraffin and were sectioned in serial transverse sections (7 μm thick) using a YD-1508R rotary microtome (Huaian Kehuai Instrument Ltd., Jiangsu, China). Dewaxed serial sections were stained with Delafield’s hematoxylin and were counterstained with eosin (HE) to show general histological characteristics.

The levels of significance in the morphological characteristics (TOL, BW, TL, HLL, HW, IL, SL, IOS and MW) and gene expression were determined by one-way analysis of variance (ANOVA) with SPSS Statistics 13.0 (SPSS Inc., Chicago, IL, USA); a value of *p* < 0.05 was considered to be statistically significant.

### Small RNA sequencing and annotation

Total RNA was extracted using Trizol (Invitrogen, Carlsbad, CA, USA) according to the manufacturer’s instructions. The total RNA quantity and purity were analyzed with Bioanalyzer 2100 (Agilent Technologies, Santa Clara, CA, USA) and RNA 6000 Nano Kit (Agilent Technologies, Santa Clara, CA, USA) with RIN number > 8.0.

After sRNAs with 15–35 nt in length were isolated from 1 μg total RNA by size fractionation in a 15% TBE urea polyacrylamide gel, the purified sRNAs were then ligated with both 3′ and 5′ adaptors for reverse transcription. After PCR amplification with forward and reverse primers complementary to the 5′ and 3′ adapters and purification with the proper size, the small RNA libraries were constructed. Subsequently, the qualified small RNA libraries were sequenced with the HiSeq 2500 platform, with 50 base-pair end-read sequencing runs (Basebio Tech, Chengdu, China). The raw data were deposited in the NCBI Sequence Read Archive (SRA) database under the study accession SRR5451121, SRR5451123, SRR5451131, SRR5451133, SRR5451135, and SRR5451136.

The raw data were processed by summarizing data production, evaluating sequencing quality, filtering low quality reads, 5′ adapter contaminants, reads without 3′ adapter, reads without insert fragment, reads containing poly (A) stretches, and reads less than 15 nt or longer than 35 bp. Length distribution analysis was performed on small RNA reads. For cataloging clean reads in terms of genome annotation to separate out rRNA, tRNA, snRNA and snoRNA, the clean RNAs were mapped to the *X. tropicalis* genome (http://www.ensembl.org), one of *M. fissipes* closely related species, by Novoalign with the maximum penalty score of alignment = 60. Subsequently, the remnant RNA sequences were re-queried against the miRNA database, miRBase 21.0 (ftp://mirbase.org/pub/mirbase/21) by using Novoalign to the further identification of conserved miRNAs. The remaining sequences that did not match known miRNAs were mapped to the *X. tropicalis* genome to identify potentially novel miRNAs by using miRDeep2. Novel miRNAs were predicted if the extended sequences at the mapped positions have the propensity of forming hairpin structures. Subsequently, we analyzed the base bias on each position of all identified conserved miRNAs in our study.

### Analysis of differentially expressed miRNAs

To determine the abundance of miRNAs and the differentially expressed miRNAs, the read counts of miRNAs were transformed into TPM (transcript per million) through the following criteria: Normalization formula: Normalized expression = (Actual miRNA count/Total count of clean reads)*1,000,000 [27]. The fold-change and *p*-value (*p* adjust) were calculated from the normalized expression using the DESeq R packages. The *p* value < 0.01 and |log2 (foldchange)| > 1 were set as the threshold for significantly differential expression.

### Target prediction and function analysis

The available RNA transcriptome data (GECV01000000) [6] was downloaded and used for the prediction of potential target mRNA candidates for significantly differently expressed miRNAs between the control and T3 groups using miRanda 3.3a [28]. To determine the function of target genes, Gene Ontology (GO) (http://www.geneontology.org/) and Kyoto Encyclopedia of Genes and Genomes (KEGG) pathway (http://www.genome.jp/kegg/) enrichment were conducted. GO annotation based on categories of biological processes, molecular functions and cellular components was performed by Blast2GO 3.1 analysis [29]. KOBAS 2.0 (http://kobas.cbi.pku.edu.cn) [30] was used to test the statistical enrichment of the target gene candidates in the KEGG pathways. In addition, because of the importance of TH signaling in anuran metamorphosis, the interaction analysis of miRNA-mRNA pairs were performed on this pathway based on the targets prediction and the expression levels of mRNAs and miRNAs. As we got the mRNA data from the previous study [6], we performed the pairwise comparison (MC vs. PM) using the DEGseq R package for screening differentially expressed genes. Unigenes were considered as differentially expressed genes at a normalized fold change > ± 2 at *p* value < 0.005 after adjustment for the false discovery rate. Afterwards, the TH pathway was downloaded from the KEGG and integration of miRNA-seq with mRNA-seq was achieved by integrating the expression profiles of differently expressed miRNAs and differently expressed mRNAs in the TH pathway with the addition of differently expressed miRNA-targeting information.

### Quantitative reverse transcriptase PCR for miRNAs

Quantitative stem-loop RT-PCR (qRT-PCR) was performed to profile the relative expression of 8 miRNAs, including 7 known and 1 novel miRNAs in the control and T3 group. Total RNA was isolated using the same reagents as described above. The expression of miRNA was detected using U6 snRNA as an endogenous control. All the primers used in the qRT-PCR are listed in Additional file 1: Table S1. The relative amount of miRNA to U6 was calculated using the 2^−ΔΔ*ct*^ method, and all quantitative data presented were the mean ± SEM.

## Results

### Morphological and histological observations in T3-induced metamorphosis

In the presence of exogenous T3, the morphology of *M. fissipes* tadpoles changed dramatically, similar to the natural metamorphosis. For example, after 48 h of T3 treatment, the tail shortened; the hindlimb buds differentiated and formed the toes; angiogenesis was observed in the hindlimb; the skin remodeled from transparent to black with pigment; the mouth became bigger; and the eyes have moved ahead (Fig. 1a). TOL, BW, TL, SL, and IOS increased after exposure to T3 at 12 h and then decreased from 24 h (Fig. 1c). Meanwhile, HLL and HW increased during T3-induced metamorphosis. MW decreased from 0 h to 36 h and then increased from 36 h to 48 h. Furthermore, TOL significantly decreased by 9.98% from 12 h to 24 h (*p* < 0.05). TOL and TL significantly decreased by 21.27 and 21.84% (*p* < 0.01) from 24 h to 36 h. Significant decreases were detected in SL (*p* < 0.01), BW (*p* < 0.05) and IOS (*p* < 0.01) at 48 h. Thus, the altered trends of these morphological characteristics after T3 treatment were similar to the natural metamorphosis (Additional file 2: Figure S1), and tadpoles exposed to 24 h of T3 treatment resembled the tadpoles at the climax of metamorphosis.

**Fig. 2:**
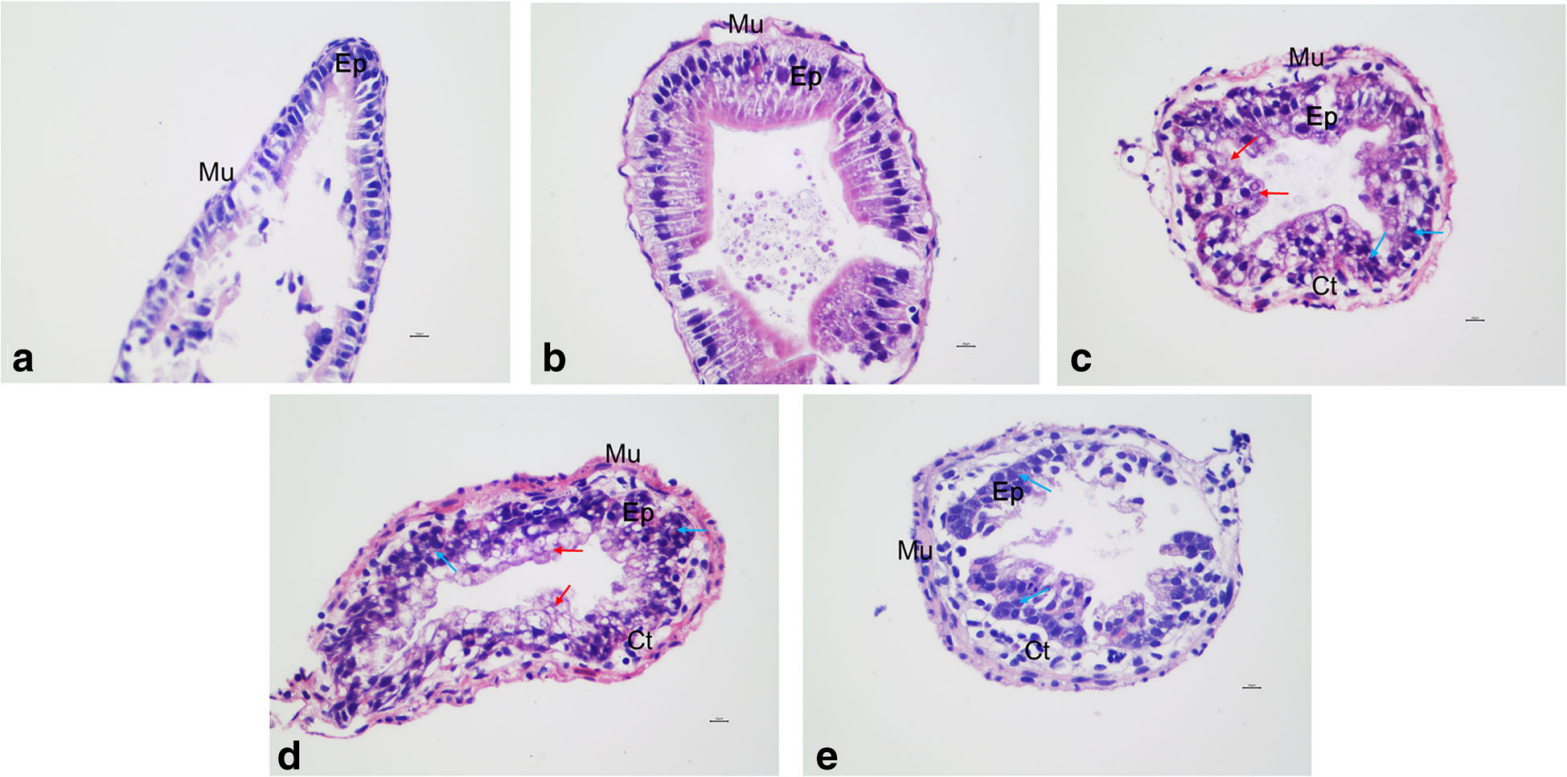
Histological changes of *M. fissipes* intestinal remodeling induced by T3. **a** control; **b** T3–12 h; **c** T3–24 h; **d** T3–36 h; **e** T3–48 h. Ct: connective tissue; Ep: epithelium; Mu: muscle. The red arrow indicates the apoptosis larval epithelial cells, while the blue arrow indicates the proliferating adult epithelial cells. Scale bar = 10 μm

In addition to external morphological changes in response to T3, we examined internal metamorphic changes. The intestine is one of the best-studied organs that remodels during metamorphosis including apoptosis of larval epithelial cells and proliferation of adult precursor epithelial cells [31]. Additionally, T3 treatment of premetamorphic *X. laevis* and *X. tropicalis* tadpoles are known to induce similar changes in the intestine to those during natural metamorphosis [32]. In our study, IL significantly decreased by 33.37, 59.86 and 72.84% at 12 h (*p* < 0.05), 36 h (*p* < 0.01) and 48 h (*p* < 0.01), respectively. To further examine the changes in the intestine during metamorphosis, the anterior small intestines from *M. fissipes* tadpoles after T3 treatment were cross-sectioned and HE stained. The premetamorphic (S33) intestine was a simple tube and contained mostly a monolayer of larval epithelial cells, with thin layers of muscles (Fig. 2a). After 12 h of T3 treatment, metamorphosis had begun, and the thin larval muscle in the intestine had begun to increase in thickness (Fig. 2b). At 24 h, larval epithelial cell death and adult epithelial cell proliferation had begun, and there was also an evident increase in the muscle and connective tissue of the intestine (Fig. 2c). At 36 h, there were numerous proliferating adult epithelial cells, apoptotic larval epithelial cells, and connective tissues (Fig. 2d). At 48 h, the epithelium developed into the multiply folded adult structure, and the connective tissue and outer muscle were abundant (Fig. 2e). T3-induced intestinal remodeling mimicked its remodeling observed during natural metamorphosis, as reported in *X. trpocalis* [32]. In particular, the *M. fissipes* tadpoles that were treated for 24 h with T3 resembled the natural tadpoles at the climax of metamorphosis. Thus, morphological and histological analysis revealed that *M. fissipes* tadpoles treated for 24 h with T3 would be selected for further miRNA-seq.

### Overview of small RNAome in *M. fissipes* metamorphosis

To survey miRNAs involved in *M. fissipes* metamorphosis, six small RNAs libraries from the control (Control-1, Control-2, Control-3) and 24 h T3 treatment (T3–1, T3–2, and T3–3) groups were constructed and sequenced. After removal of the adapters, low-quality reads, reads with unknown nucleotides, reads smaller than 15 nucleotides, reads larger than 35 nucleotides, reads without a 3′ adaptor, and reads with polyA/T, 17.2, 19.1, 14.2, 18.6, 15.3, and 18.8 million clean reads were generated in the Control-1, Control-2, Control-3, T3–1, T3–2, and T3–3 libraries, respectively (Additional file 3: Table S2). An overview of the reads for sRNA-seq from the raw data to high-quality data and with quality filtering is shown in Additional file 3: Table S2.

The length distributions of sRNAs were similar between two groups in which 22 nt RNAs were the most abundant (Additional file 4: Figure S2a). After genome annotation to separate out rRNA, tRNA, snRNA, snoRNA and mRNA, more than 6 million sequences corresponding to 70.11 and 65.40% of all clean reads were annotated to known miRNAs in the control and T3 treatment group, respectively (Additional file 5: Figure S3). These results showed that our sRNA libraries were highly enriched with mature miRNAs.

### Identification of conserved and novel miRNAs in *M. fissipes* metamorphosis

Many miRNAs have been conserved during evolution, and the sequence identity is extremely high even among evolutionarily distant species [33, 34]. To investigate the conserved miRNAs in *M. fissipes* metamorphosis, the sequences of the miRNAs from the six libraries were compared to *X. tropicalis* miRNA sequences in miRBase 21.0. In total, 164 conserved miRNAs belonging to 82 miRNA families in the libraries (Additional file 6: Table S3) were identified. The details families of the member numbers of conserved miRNAs are summarized in Additional file 6: Table S3. A total of 34 conserved miRNA families contained more than one member.

The conserved miRNAs have a broad range of expression levels, varying from 1 count to 978,287 counts, confirming the thoroughness of our miRNA screen strategy. For T3 and control libraries, the majority of unique sequences for conserved miRNAs exhibited high abundance (> 1000 counts), although a few unique sequences were present at less than 10 counts (Additional file 4: Figure S2c). Among the miRNAs with high abundance (more than 100,000 counts), 12 miRNAs (mfi-miR-192, mfi-miR-26, mfi-miR-143, mfi-miR-148a, mfi-miR-205a, mfi-miR-22-3p, mfi-miR-181a-5p, mfi-miR-182-5p, mfi-miR-194, mfi-miR-200a, mfi-miR-92a, and mfi-let-7f) were most highly expressed in *M. fissipes* metamorphosis. In addition, 7 members of the let-7 family (including mfi-let-7a, − 7b, − 7c, −7e, −7f, −7 g, and -7i) were expressed in metamorphosis and their responses to T3 were different.

The mature miR-#-5p and miR-#-3p are pairwise miRNAs that align to the 5′ and 3′ end regions of the same precursors, respectively. In *M. fissipes*, 15 pairs of known miR-#-5p and miR-#-3p were detected. Among these, 10 pairwise miRNAs (mfi-miR-142, mfi-miR-17, mfi-miR-18a, mfi-miR-20a, mfi-miR-181a, mfi-miR-182, mfi-miR-199a, mfi-miR-30a, mfi-miR-9a, and mfi-miR-9b) were found to have relatively lower expression levels of miR-#-3p than their miR-#-5p counterparts, while the other 5 pairwise miRNAs (miR-29c, miR-22, miR-363, miR-24a, and miR-126) showed relatively higher expression levels of miR-#-3p.

Furthermore, one of the greatest advantages of high-throughput sequencing is that this technology can be used to identify novel miRNAs. In this study, 36 novel miRNAs were predicted. In addition, all novel miRNAs were found in all libraries.

The composition patterns of miRNAs are closely correlated with the biological characteristics of different development stages. The nucleotide bias on each position of all identified miRNAs is shown in Additional file 4: Figure S2b. The majority of miRNAs tended to start with 5’-U but not 5′-G in *M. fissipes*. Interestingly, the 5′ ninth nucleotide was also significantly enriched in U in our study.

### Identification and validation of miRNAs differentially expressed in *M. fisssipes* metamorphosis

A total of 21 differentially expressed miRNAs were identified between the control group and T3 treated group (|log2foldchange| > 1, *p* < 0.01) (Additional file 4: Figure S2d), including 20 known miRNAs (mfi-miR-10a, mfi-miR-10b, mfi-miR-133c, mfi-miR-148b, mfi-miR-15a, mfi-miR-15b, mfi-miR-17-5p, mfi-miR-181b, mfi-miR-199a-5p, mfi-miR-216, mfi-miR-222, mfi-miR-25, mfi-miR-301, mfi-miR-30d, mfi-miR-30e, mfi-miR-31a, mfi-miR-363–3p, mfi-miR-429, mfi-miR-9a-5p, and mfi-miR-9b-5p) and one novel miRNA (novel-miR-10).

**Fig. 3:**
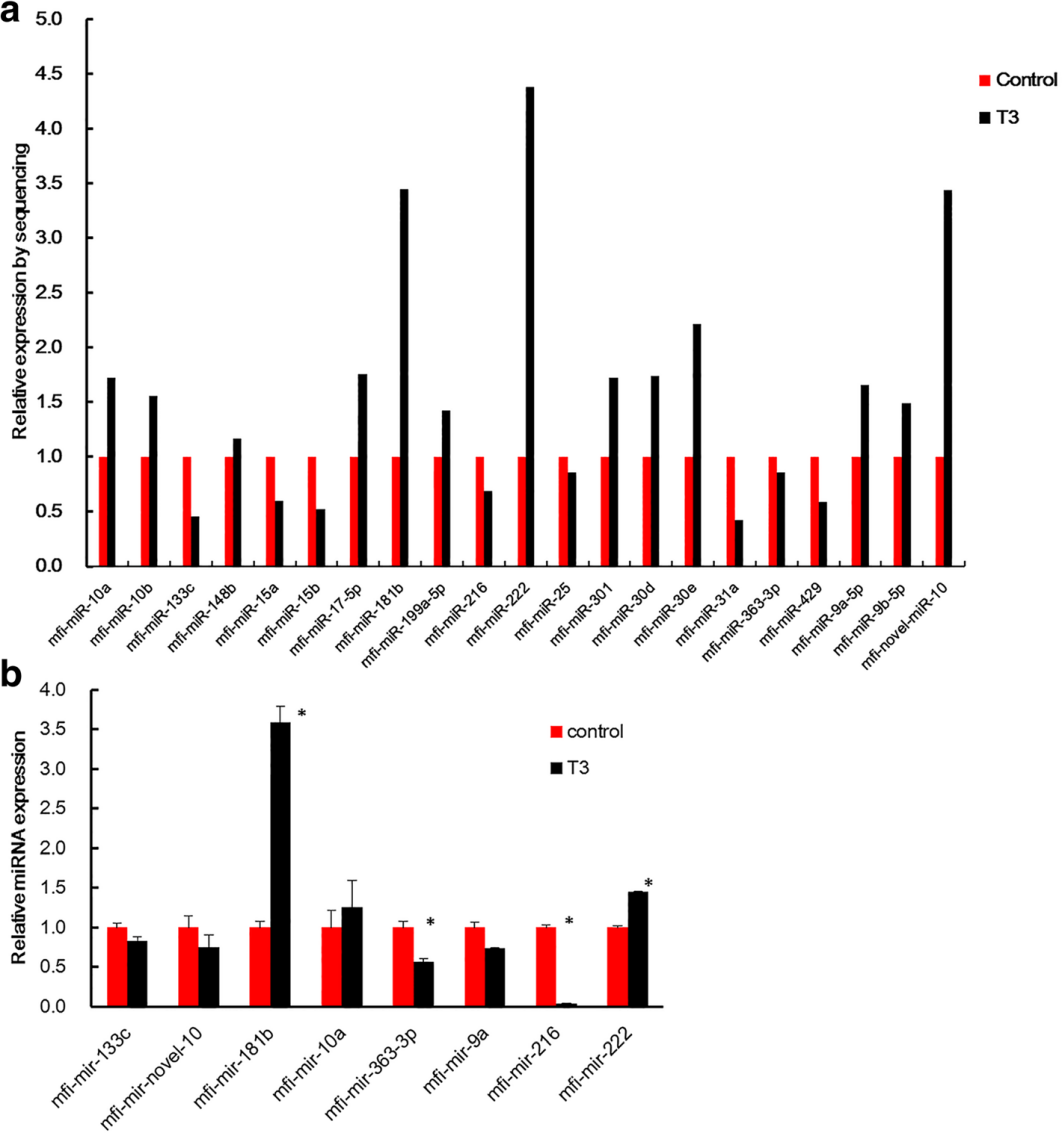
Differential expression levels of miRNAs in the control and T3 groups. **a** Significantly different expression miRNAs in the two groups based on deep sequencing data. **b** Validation of miRNAs by qRT-PCR, * means a statistically significant difference (*p* < 0.05) between the T3 and control groups; y-axis means the Fold change with control group as 1

Among these, the expression levels of 8 and 13 miRNAs were suppressed and promoted by T3 treatment, respectively (Fig. 3a). To validate the expression patterns of miRNAs by deep sequencing, 8 miRNAs with significant expression changes were randomly selected for qRT-PCR analysis. Except novel-miR-10, the expression patterns obtained by qRT-PCR for the control and T3 groups were highly consistent with the sequencing results (Fig. 3b). These results indicated that small-RNA sequencing is a sensitive and reliable method to identify differentially expressed miRNAs in *M. fissipes* metamorphosis.

### Target prediction of differentially expressed miRNAs and function analysis in *M. fissipes* metamorphosis

The functions of miRNAs on gene regulation mainly rely on the interaction between miRNAs and their target genes via complementation [35]. Therefore, identification of miRNA target genes is essential to understand the regulatory function of miRNA. Target prediction was performed against the unigenes from the *M. fissipes* transcriptome using miRanda (3.3a). A total of 10,206 unigenes (out of 26,323) were predicted as potential target genes of miRNAs in this study. It indicated that each miRNA always corresponded to hundreds of target genes. For example, mfi-miR-181b could bind to collagen genes, including *col5a3*, *col6a3*, and *col12a1*; ECM proteolysis related genes, including *matrix metalloproteinase 2* and *matrix metalloproteinase 14*; and the hedgehog target gene *twist2*. All of these genes were involved in natural metamorphosis during the climax of metamorphosis stage for patterning [6]. Additionally, a protein coding gene may also be targeted by multiple miRNAs at different targeting sites. For example, *TRα*, which is essential for metamorphosis initiation in *M. fissipes*, was predicted as the target of 7 miRNAs including mfi-miR-10a, mfi-miR-10b, mfi-miR-148b, mfi-miR-15b, mfi-miR-31a, mfi-miR-9a-5p, and mfi-miR-9b-5p. Therefore, many predicted targets were likely to be regulated by multiple miRNAs at different targeting sites, while miRNAs targeted to different genes at different sites.

**Fig. 4:**
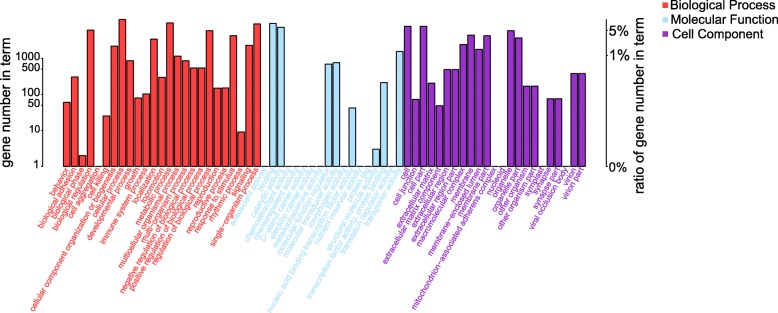
Gene ontology (GO) classification of differentially expressed target genes

**Fig. 5:**
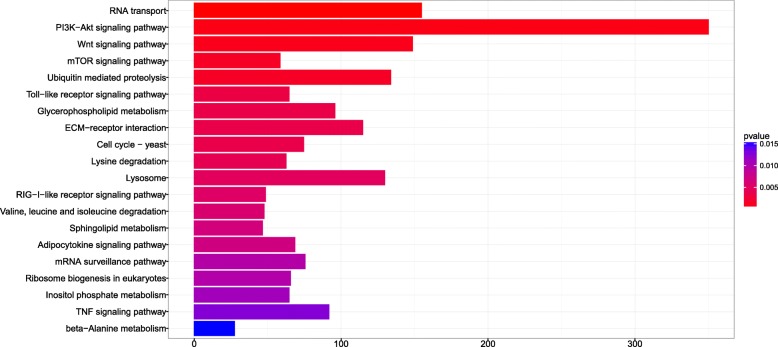
KEGG pathway analysis of differently expressed target genes. The top 20 significant enriched pathways based on KEGG analysis. The y-axis shows 20 different enriched pathways in which the target gene productions are involved in. The x-axis shows the number of genes enriched in each pathway

To obtain an overview of the pathways in which the miRNAs were involved, the predicted target genes for the differentially expressed miRNAs were subjected to GO analysis and KEGG pathway analysis. GO enrichment analysis showed that 216 cellular component ontology terms, 134 molecular function ontology terms and 704 biological process ontology terms were highlighted (Fig. 4). Similarly, all predicted target genes were also blasted against the KEGG database, and 251 pathways were highlighted (*p* < 0.01), suggesting that each pathway included several miRNAs. The top twenty enriched pathways are shown in Fig. 5. Interestingly, the most overrepresented miRNA target genes were enriched in the “PI3k-Akt signaling pathway”. In addition, RNA transport, ECM-receptor interaction, Wnt signaling pathway, mTOR signaling pathway, ubiquitin mediated proteolysis and lysosome were also significantly enriched.

### Network of putative interactions in the TH pathway

**Fig. 6:**
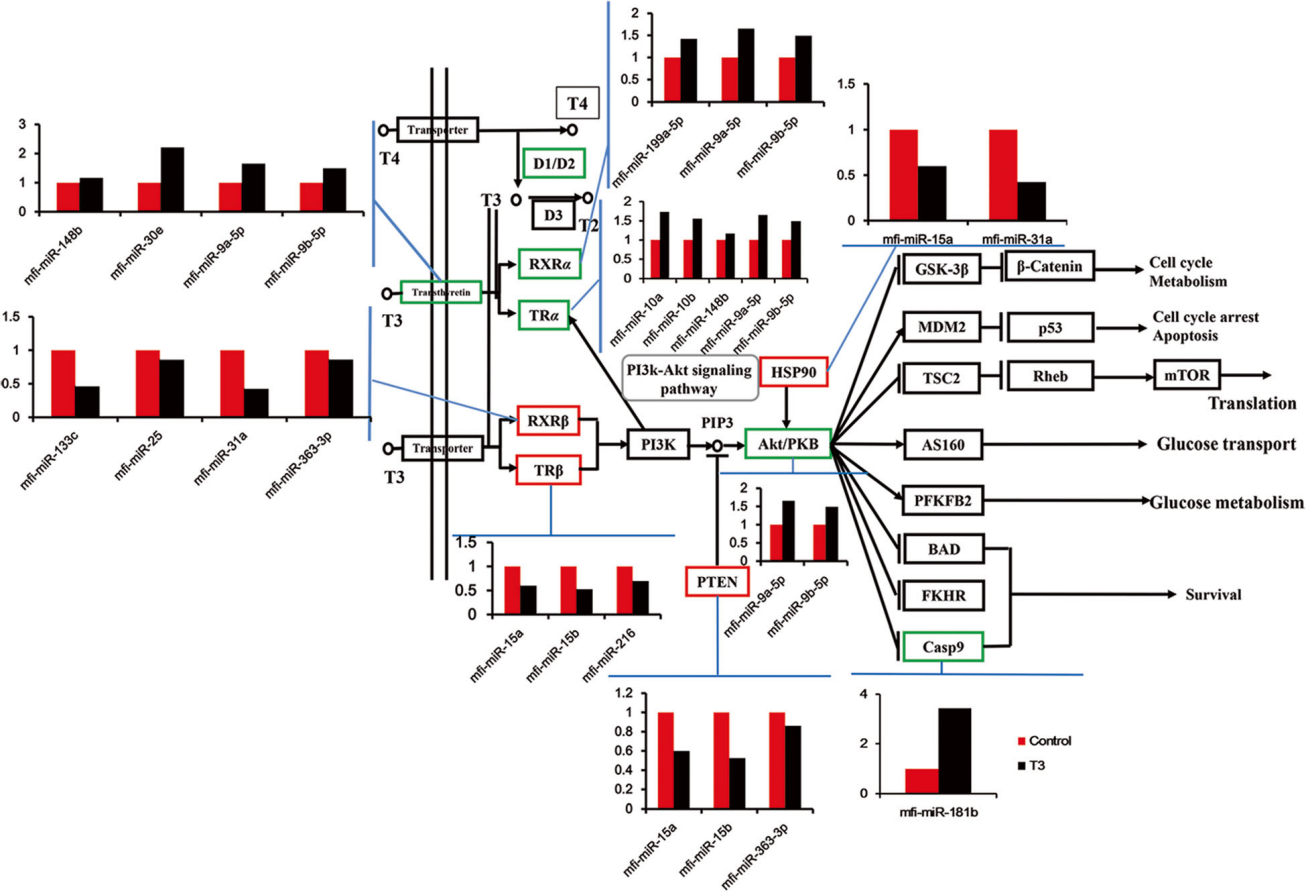
A network of putative interactions between differently expressed miRNAs and target genes in the TH signaling pathway. The red box indicates the genes that were upregulated by T3 treatment, while the green box indicates the genes that were downregulated by T3. The blue line presented the relative expression of miRNAs targeting the gene

Since a single miRNA can target multiple mRNAs and one mRNA can also be targeted by multiple miRNAs, a context-specific network of miRNA:mRNA interactions can be illuminated. Based on the data from RNA-seq and the importance of the TH signaling pathway in the metamorphosis, differentially expressed miRNAs and their target genes that showed inverse expression patterns were co-mapped in the TH signaling pathway (Fig. 6). MiRNAs and their target genes with opposite counts during metamorphosis were considered potential interactions.

Among the 10 significantly differential expressed target genes involved in the TH signal pathway, 4 genes (*HSP90*, *PTEN*, *RXRβ* and *TRβ*) were upregulated from premetamorphosis to the climax of metamorphosis, while *TRα*, *RXRα*, *type I iodothyronine deiodinase* (*D1*) and *transthyretin* were downregulated. It was reported that *TRα*-deficient tadpoles develop faster, however, with a smaller body size at the same stage than their wild-type siblings, suggesting that *TRα* plays an important role in controlling the timing of metamorphosis [36, 37]. In addition, mfi-miR-10a, mfi-miR-10b, mfi-miR-148b, mfi-miR-9a-5p and mfi-miR-9b-5p, which are predicted to target *TRα*, were up-regulated. *RXRβ* and *TRβ*, which initiated the process of metamorphosis, were up-regulated, while their predicted interaction miRNAs (mfi-miR-133c, mfi-miR-25, mfi-miR-31a and mfi-miR-363–3p; mfi-miR-15a, mfi-miR-15b and mfi-miR-216) were down-regulated. We also obtained the interactions of miRNAs and genes from the PI3k-Akt signaling pathway which has been enriched in the KEGG analysis. The expression of *Akt* decreased during metamorphosis, while the expression of mfi-miR-9a-5p and mfi-miR-9b-5p, which target Akt increased. *Casp9*-mfi-miR-181b interaction was also detected, indicating that miRNA regulated anuran metamorphosis by posttranscriptional regulation of genes in PI3k-Akt signaling pathway and in the TH signaling pathway. Therefore, synergistic regulatory pathways among multiple miRNAs and mRNAs play important roles in anuran metamorphosis.

## Discussion

In anuran, the transition from larval to juvenile is controlled directly by steadily rising levels of TH. The rising level of TH initiated the transcription of immediate response genes at varying thresholds in each responding tissue and led to extensive morphological and physiological remodeling of tissues. Treated with T3, *M. fissipes* prematurely accelerated development toward the metamorphic climax similar to that of *X. tropicalis* [31]. Treatment of *M. fissipes* tadpoles for 24 h with T3 resulted in the similar of morphological and histological characteristics of natural tadpoles at the climax of metamorphosis. Therefore, the control group and the 24 h T3 treatment group were selected for miRNA-seq to screen and identify TH-responding miRNAs and to reveal the integrated regulation of TH during *M. fissipes* metamorphosis. Furthermore, due to the near total dependence of anuran metamorphosis on TH, *M. fissipes* provides an excellent model to develop an assay for TH system disruption across all vertebrates.

Few miRNAs have been reported to be involved in anuran metamorphosis [24]. Using the high-throughput sequencing approach, we identified 164 conserved miRNAs and 36 novel miRNAs and analyzed the expression profiles of these miRNAs during *M. fissipes* metamorphosis. The most-abundant size class of sRNAs was 22 nt, followed by 23 and 21 nt. This was consistent with the known size of Dicer-processed small RNAs in *X. tropicalis* somatic libraries and other organisms [20, 38, 39]. The expression levels of all miRNA sequences ranged from 1 to 978,287 counts, indicating that not only miRNAs with high expression but also miRNAs with low expression were detected by Illumina sequencing. Therefore, Illumina sequencing is a more efficient and accurate approach than the traditional cloning method to identify conserved and novel miRNAs.

The expression levels of mfi-miR-192, mfi-miR-26, mfi-miR-143, mfi-miR-148a, mfi-miR-205a, mfi-miR-22-3p, mfi-miR-181a-5p, mfi-miR-182-5p, mfi-miR-194, mfi-miR-200a, mfi-miR-92a, and mfi-let-7f were highest in this study, implying their potential significant functions in *M. fissipes* metamorphosis. Intriguingly, the number of miR-143a clones was almost one third of the total number of cloned miRNAs during *X. tropicalis* metamorphosis [24], suggesting its certain conserved role in supporting metamorphosis. MiR-143, miR-181a-5p and miR-22-3p were highly enriched in skeletal muscle tissue [40, 41], which was the major tissue of *M. fissipes* tadpoles for small RNA-seq. MiR-92 was reported to play a regulated role in the proliferation of myeloid cells [42], while miR-192 suppressed cell proliferation and induced apoptosis. High expression of these two miRNAs in *M. fissipes* tadpoles further confirmed that their roles in proliferation, differentiation and apoptosis during anuran metamorphosis [40, 41, 43]. MiR-182 was significantly upregulated in anaplastic thyroid cancer (ATC) tissues and cells [44]; its high abundance in this study may suggest its role in thyroid cell activation during metamorphosis. Different expression levels of the let-7 family members implied that the let-7 family likely conducted precise regulation of different target genes, and let-7 miRNAs have been reported to widely participate in tissue development and metabolism during development and the regulation of temporal transitions associated with cell proliferation and differentiation during Japanese flounder metamorphosis [45]. Furthermore, let-7 family miRNAs have indeed been reported to be involved in numerous cellular processes, including as master regulators of the cell cycle [46, 47], indicating that the let-7 family was likely critical to morphological reorganization during metamorphosis by regulating the cell cycle.

Different expression levels between miR-#-5p and their miR-#-3p counterparts were detected in 15 pairs of miRNAs during *M. fissipes* metamorphosis. Although both strands of the miRNA duplex were equally produced after the transcription of the miRNA genes, their stability in the the single-stranded form was different [48]. As a result, the expression levels of miR-#-3p/5p mainly relied on their respective degradation degrees and degradation rates [27]. Some miR-#-3 ps were reported as mature functional miRNAs with abundant expression, and miR-#-3p/miR-#-5p ratios may vary dramatically in different stages of development [49]. On the other hand, it was found that most of the miR-#-3 ps were detected at the same level or at relatively lower expression levels than miR-#-5p [27, 50, 51]. In this study, more miR-#-5 ps with higher expression levels indicated their importance in regulating gene expression.

The 3′ and 5′ edges of the ‘seed region’, which were known to have a critical role in targeting miRNA to mRNA for translational inhibition or mRNA cleavage were both flanked by U, which has been detected in other vertebrates [46]. This phenomenon suggested that the U base may have effects on miRNA action, such as miRNA biogenesis, mRNA target recognition and miRNA binding to targets for gene regulation. High conservation was also observed at the first position upstream and downstream of the seed match in the target gene. This nucleotide was often a conserved A, which could pair to the nucleotide of the miRNA with 5′ and 3′ edges of its ‘seed region’ were U [52].

In total, 20 known miRNAs (mfi-miR-10a, mfi-miR-10b, mfi-miR-133c, mfi-miR-148b, mfi-miR-15a, mfi-miR-15b, mfi-miR-17-5p, mfi-miR-181b, mfi-miR-199a-5p, mfi-miR-216, mfi-miR-222, mfi-miR-25, mfi-miR-301, mfi-miR-30d, mfi-miR-30e, mfi-miR-31a, mfi-miR-363–3p, mfi-miR-429, mfi-miR-9a-5p and mfi-miR-9b-5p) and one novel miRNA (novel-miR-10) were found to be differentially expressed between the control and T3 groups, indicating that these miRNAs might play important roles in diverse biological processes that resulted in the transition from larval to juvenile *M. fissipes*. MiR-222 and miR-181b are potential molecular markers used to differentiate papillary thyroid cancer from normal thyroid tissue [53], suggesting their potential roles in regulating thyroid cell division and differentiation during metamorphosis. Interestingly, T3 treatment activated the expression of these two miRNAs in *M. fissipes* tadpoles. It seems that miR-222 and miR-181b are involved in a positive feedback cycle in amplifying the TH signal to achieve the dramatic metamorphosis. MiR-133 is a muscle-specific miRNA reported to positively regulate differentiation and proliferation of myoblasts [54]. Our study showed that the expression of mfi-miR-133c was suppressed by T3 treatment. Given that replacement and conversion between different types of myofilaments was one of the most dramatic changes during anuran metamorphosis [43], down-regulation of mfi-miR-133c may be necessary to suppress the expression of tadpole-type myofilaments.

The impact of these 21 microRNAs is amplified by a larger number of predicted target genes. Therefore, identification of miRNA targets is an important step to thoroughly describing the function of miRNAs. Further investigation of these differentially expressed miRNAs would be helpful to explain the complex genetic network that controls processes of metamorphosis and to provide insight into metamorphic changes. Based on the principle of high miRNA-target complementarity, 10,206 target genes were predicted. It was currently estimated that the human miRNAs targeted ∼ 60% of all protein-coding genes [55], indicating that each miRNA always corresponded to hundreds of target genes. GO and KEGG pathway analysis showed that the predicted target genes were involved in diverse biological processes, especially cellular operations. These results showed that miRNAs participated in metamorphosis by targeting genes in these pathways. Intriguingly, the most significantly enriched pathway targeted by the differentially expressed miRNAs was the PI3k-Akt signaling pathway, which is the central signaling pathway responding to hormones, extracellular signal molecules and nutrients to regulate numerous cellular events such as metabolic homeostasis, proliferation, growth, and survival [56]. Pathways associated with RNA transport, the Wnt signaling pathway, the mTOR signaling pathway, ubiquitin-mediated proteolysis and lysosome-related pathways were also significantly enriched, indicating the role of differentially expressed miRNAs in the regulation of cell growth, cell proliferation, and apoptosis. Moreover, the enrichment of cell-cell and cell-ECM interactions suggested that the differentially expressed miRNAs participated in communication between cells and the extracellular matrix, and then affected cellular changes, including apoptosis, cell proliferation, migration and differentiation [57]. Ubiquitin -mediated proteolysis and lysosomal degradation occur during metamorphosis, where massive breakdown of larval tissue occurs [58], thus explaining the enrichment of miRNA-targeted genes in the ubiquitin-mediated proteolysis and lysosome degradation pathway. Furthermore, miRNAs enriched in the Wnt signaling pathway are necessary for proper formation of important tissues [59], such as the hindlimb, adult dorsal muscle, adult intestine and adult liver. Therefore, the enrichment results indicated that these differentially expressed miRNAs participated in regulating cell growth, proliferation, communication and apoptosis during *M. fissipes* metamorphosis.

Based on the integrated miRNA and mRNA analysis together with target prediction and the importance of the TH signaling pathway during metamorphosis, a network of differentially expressed miRNAs and differentially expressed genes in the TH pathway was constructed. The expression of miR-10a was switched on by *RARα*, a hormone receptor modulated by *RXRα* [60]. Because *RXRα* and *TRα* were upregulated by T3 during metamorphosis and *TRα* was the target gene of miR-10a, T3-*RXRα-RARα-*miR-10a-*TRα* played the negative feedback role in *M. fissipes* metamorphosis. Elevated expression of *TRα* and decreased mfi-miR-10a, mfi-miR-10b, mfi-miR-148b, mfi-miR-9a-5p and mfi-miR-9b-5p in the pathway indicated that these miRNAs were essential for the initiation of metamorphosis in *M. fissipes*. It also implied that *TRα* is not only the receptor mediated by TH signals but is also a potential target gene regulated by miRNA. It was important for future studies to functionally validate the predicted function of these differentially expressed miRNAs in this pathway.

## Conclusion

Our study is the first study on the miRNAs in *M. fissipes* metamorphosis and the integrated analysis of mRNA-seq and miRNA-seq in amphibians. This study provides an overview of the miRNAs expressed in TH-dependent metamorphosis in *M. fissipes* through the use of high-throughput Illumina sequencing technology. In total, 164 conserved and 36 predicted novel miRNAs were identified in the *M. fissipes* metamorphosis. Of those, 21 differentially expressed miRNAs were detected in two metamorphic groups, and functional annotation showed that the differentially expressed miRNAs along with their target genes might be essential to cell growth, cell proliferation, communication and apoptosis. This study provided the basis for future analysis of miRNA function in *M. fissipes* metamorphic development and gave us clues for further studies of the mechanisms of *M. fissipes* metamorphosis. Furthermore, using *M. fissipes* as the model organism, the miRNA detected in this study may provide a marker for the identification of environmental chemical contaminants that perturb TH signaling in vertebrates.

## Additional files

Additional file 1: **Table S1.** Primers sequences for qRT-PCR. (DOC 38 kb)

Additional file 2: **Figure S1.** Morphological characteristics of *M. fissipes* tadpoles during natural metamorphosis. TOL: total length; TL: tail length; BW: body width; SL: snout length; IOS: interocular space; Pre-: premetamorphosis; Pro-: prometamorphosis; climax: the climax of metamorphosis; End: end of metamorphosis. (TIF 761 kb)

Additional file 3: **Table S2.** Overview of readcounts for sRNA-seq from the raw data to high quality reads, and quality filtering. (DOC 42 kb)

Additional file 4: **Figure S2.** a. Length distribution and abundance of small RNA sequences in *M. fissipes*, as determined by Illumina small-RNA deep sequencing. b. Nucleotides bias on the specific position of miRNAs in *M. fissipes* c. Count distribution and abundance of unique small RNA sequences in *M. fissipes*. d. Scatter plot map for miRNAs expression in the control and T3 groups. Each plot represented an individual miRNA, while the red plot indicated the significantly differentially expressed miRNA (*p* < 0.01 and |log2 (foldchange)| > 1). (JPG 1976 kb)

Additional file 5: **Figure S3.** Pie charts of different abundance of small RNA in control group and T3 treated group. (TIF 579 kb)

Additional file 6: **Table S3.** Conserved miRNAs in *M. fissipes*. (XLS 61 kb)

## Abbreviations

BW: body width
GO: gene ontology
HLL: hindlimb length
HW: hindlimb width
IL: intestine length
IOS: interocular space
KEGG: Kyoto Encyclopedia of Genes and Genomes
MW: mouth width
qRT-PCR: quantitative reverse transcriptase PCR
RXR: 9-cis-retinoic acid receptor
SL: snout length
TH: thyroid hormone
TL: tail length
TOL: total length
TR: thyroid hormone receptors
UTR: untranslated region
