# Supplementary material for: Identification and differential regulation of microRNAs during thyroid hormone-dependent metamorphosis in Microhyla fissipes
Source: BMC Genomics. 2018 Jun 28;19:507. doi: 10.1186/s12864-018-4848-x (PMC6025837; doi:10.1186/s12864-018-4848-x)
Supplement: Supplementary file 1 — Table S1. Primers sequences for qRT-PCR. (DOC 38 kb) [file 12864_2018_4848_MOESM1_ESM.doc]

**Table S1.** Primers sequences for qRT-PCR.

| Primer Name | Specific Primer Sequence (5’-3’) |
| --- | --- |
| U6-S | CTCGCTTCGGCAGCACA |
| U6-A | AACGCTTCACGAATTTGCGT |
| Universal-A | TGGTGTCGTGGAGTCG |
| mfi-miR-181b-RT | CTCAACTGGTGTCGTGGAGTCGGCAATTCAGTTGAGAACCCACC |
| mfi-miR-181b-S | ACACTCCAGCTGGGAACATTCATTGCTGTCGG |
| mfi-miR-9a-RT | CTCAACTGGTGTCGTGGAGTCGGCAATTCAGTTGAGTCATACAG |
| mfi-miR-9a-S | ACACTCCAGCTGGGTCTTTGGTTATCTAGCT |
| mfi-miR-222-RT | CTCAACTGGTGTCGTGGAGTCGGCAATTCAGTTGAGGAGACCCA |
| mfi-miR-222-S | ACACTCCAGCTGGGAGCTACATCTGGCTACTG |
| mfi-miR-216-RT | CTCAACTGGTGTCGTGGAGTCGGCAATTCAGTTGAGCACAGTTG |
| mfi-miR-216-S | ACACTCCAGCTGGGTAATCTCAGCTGGCA |
| mfi-miR-133c-RT | CTCAACTGGTGTCGTGGAGTCGGCAATTCAGTTGAGCAGCTGGT |
| mfi-miR-133c-S | ACACTCCAGCTGGGTTGGTCCCCTTCAAC |
| mfi-miR-10a-RT | CTCAACTGGTGTCGTGGAGTCGGCAATTCAGTTGAGACAAATTC |
| mfi-miR-10a-S | ACACTCCAGCTGGGTACCCTGTAGATCCGA |
| mfi-miR-363-3p-RT | CTCAACTGGTGTCGTGGAGTCGGCAATTCAGTTGAGACAGATGG |
| mfi-miR-363-3p-S | ACACTCCAGCTGGGAATTGCACGGTATCC |
| miR-novel-10-RT | CTCAACTGGTGTCGTGGAGTCGGCAATTCAGTTGAGCAGGATTC |
| miR-novel-10-S | ACACTCCAGCTGGGCCCCGCGCAGGTTCGA |
