# Supplementary figures and images for: Identification and differential regulation of microRNAs during thyroid hormone-dependent metamorphosis in Microhyla fissipes
Source: BMC Genomics. 2018 Jun 28;19:507. doi: 10.1186/s12864-018-4848-x (PMC6025837; doi:10.1186/s12864-018-4848-x)

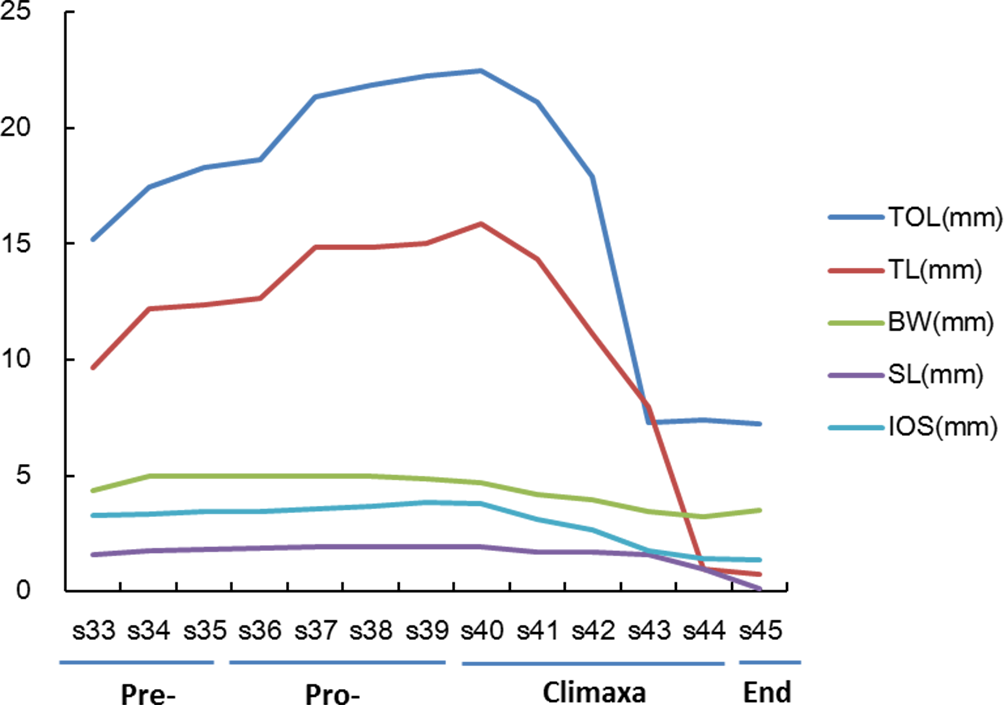

Supplement: Supplementary file 2 — Figure S1. Morphological characteristics of M. fissipes tadpoles during natural metamorphosis. TOL: total length; TL: tail length; BW: body width; SL: snout length; IOS: interocular space; Pre-: premetamorphosis; Pro-: prometamorphosis; climax: the climax of metamorphosis; End: end of metamorphosis. (TIF 761 kb) [file 12864_2018_4848_MOESM2_ESM.tif]

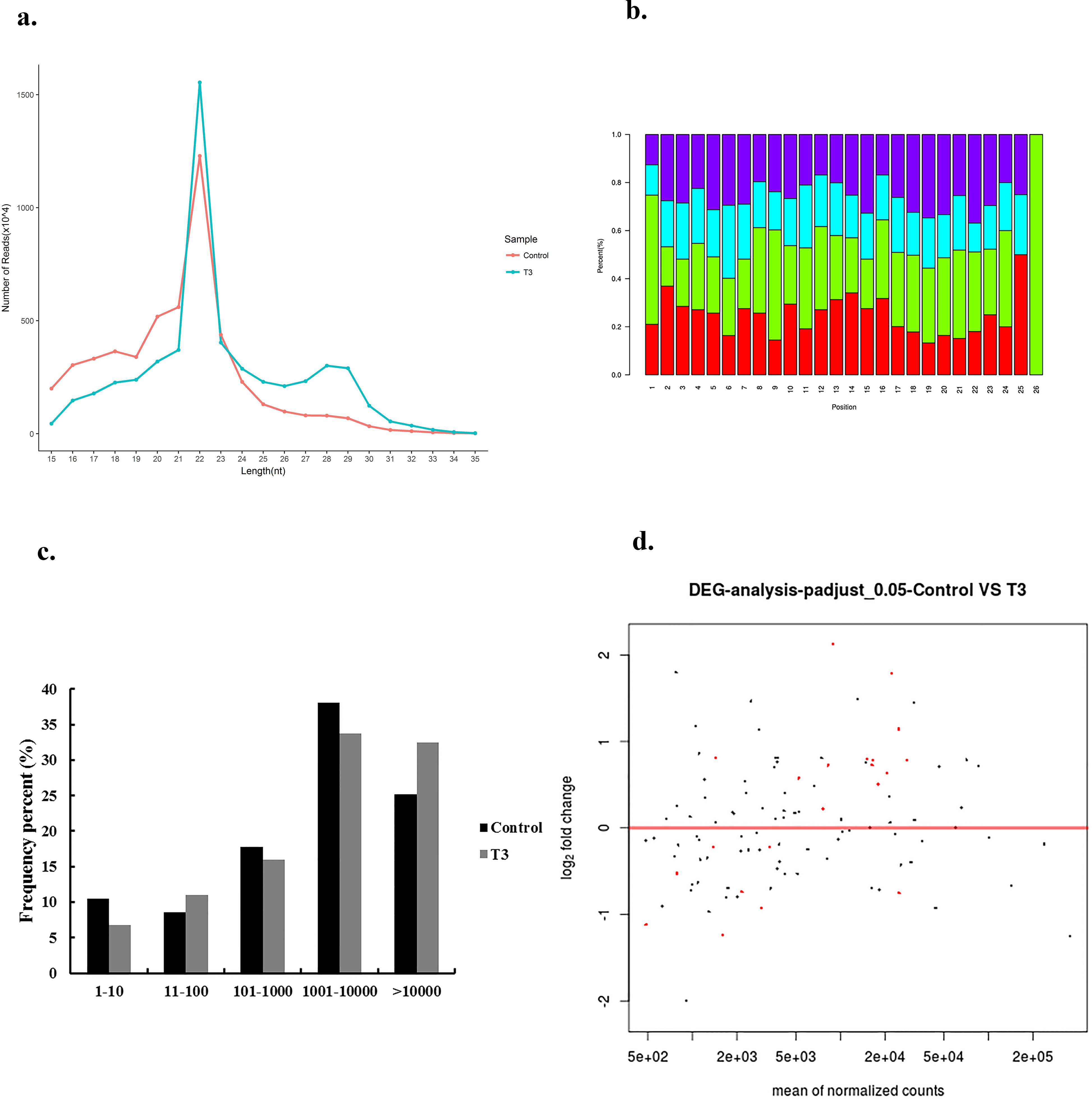

Supplement: Supplementary file 4 — Figure S2. a. Length distribution and abundance of small RNA sequences in M. fissipes, as determined by Illumina small-RNA deep sequencing. b. Nucleotides bias on the specific position of miRNAs in M. fissipes c. Count distribution and abundance of unique small RNA sequences in M. fissipes. d. Scatter plot map for miRNAs expression in the control and T3 groups. Each plot represented an individual miRNA, while the red plot indicated the significantly differentially expressed miRNA (p < 0.01 and |log2 (foldchange)| > 1). (JPG 1976 kb) [file 12864_2018_4848_MOESM4_ESM.jpg]

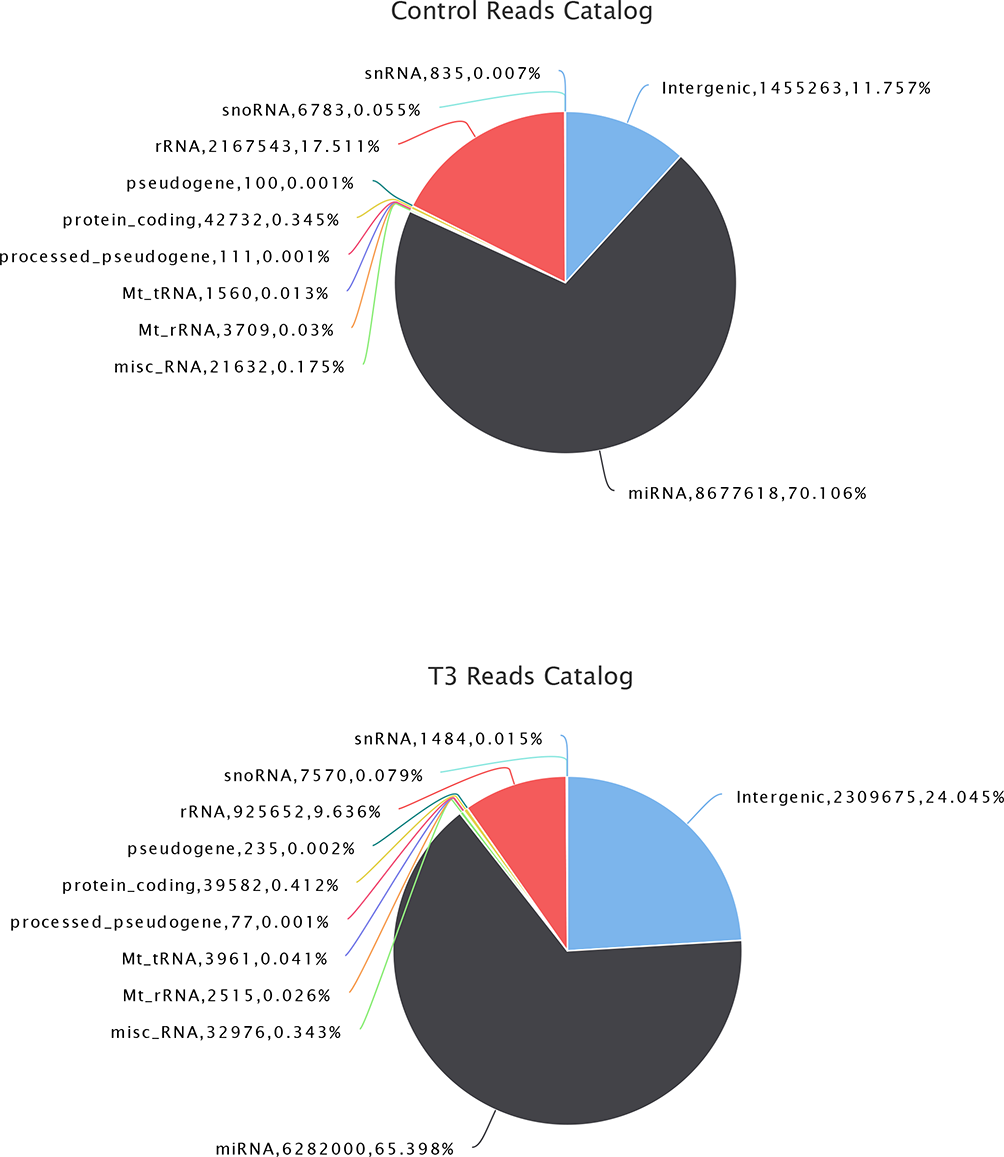

Supplement: Supplementary file 5 — Figure S3. Pie charts of different abundance of small RNA in control group and T3 treated group. (TIF 579 kb) [file 12864_2018_4848_MOESM5_ESM.tif]
