# Supplementary material for: Identification and differential regulation of microRNAs during thyroid hormone-dependent metamorphosis in Microhyla fissipes
Source: BMC Genomics. 2018 Jun 28;19:507. doi: 10.1186/s12864-018-4848-x (PMC6025837; doi:10.1186/s12864-018-4848-x)
Supplement: Supplementary file 3 — Table S2. Overview of readcounts for sRNA-seq from the raw data to high quality reads, and quality filtering. (DOC 42 kb) [file 12864_2018_4848_MOESM3_ESM.doc]

**Table S2.** Overview of readcounts for sRNA-seq from the raw data to high quality reads, and quality filtering.

| Sample | Control-1 | Control-2 | Control-3 | T3-1 | T3-2 | T3-3 |
| --- | --- | --- | --- | --- | --- | --- |
| Total Raw Reads | 23806332 | 23802831 | 23206822 | 23763764 | 21470607 | 22047764 |
| Total Raw Bases | 1190316600 | 1190141550 | 1160341100 | 1188188200 | 1073530350 | 1102388200 |
| Total Clean Reads | 17158111 | 19054845 | 14235242 | 18615824 | 15346019 | 18807319 |
| Total Clean Bases | 348914820 | 418322293 | 290099791 | 400838425 | 336359445 | 473332253 |
| Unique Clean Reads | 1983353 | 1459021 | 2080251 | 1800199 | 698619 | 2172182 |
| Clean Reads Rate (%) | 72.07 | 80.05 | 61.34 | 78.34 | 71.47 | 85.3 |
| Low Quality Reads | 6479 | 6897 | 6464 | 7975 | 4473 | 9598 |
| Low Quality Rate (%) | 0.03 | 0.03 | 0.03 | 0.03 | 0.02 | 0.04 |
| Without 3p Adaptor Reads | 1652368 | 1547767 | 1688041 | 1552958 | 1246894 | 1411822 |
| Without 3p Adaptor Rate (%) | 6.94 | 6.5 | 7.27 | 6.53 | 5.81 | 6.4 |
| Without Insert Reads | 20199 | 10504 | 12743 | 9241 | 79991 | 6115 |
| Without Insert Rate (%) | 0.08 | 0.04 | 0.05 | 0.04 | 0.37 | 0.03 |
| Polya/T Reads | 2793721 | 2021433 | 2360199 | 1646342 | 451135 | 905804 |
| Polya/T Rate (%) | 11.74 | 8.49 | 10.17 | 6.93 | 2.1 | 4.11 |
| Ex-Length Reads | 2175454 | 1161385 | 4904133 | 1931424 | 4342095 | 907106 |
| Ex-Length Rate (%) | 9.14 | 4.88 | 21.13 | 8.13 | 20.22 | 4.11 |
| Raw Reads Q20 (%) | 97.56 | 97.62 | 97.46 | 97.77 | 97.75 | 97.61 |
| Raw Reads Q30 (%) | 95.07 | 95.26 | 94.86 | 95.51 | 95.36 | 95.13 |
| Clean Reads Q20 (%) | 98.25 | 98.19 | 98.07 | 98.07 | 98.05 | 98.14 |
| Clean Reads Q30 (%) | 96.03 | 95.94 | 95.61 | 95.64 | 95.65 | 95.75 |
